# Evaluation of 4-hand reduction for obturator hernia with the guidance of sonography as a new treatment strategy: A retrospective study

**DOI:** 10.1097/MD.0000000000031375

**Published:** 2022-10-28

**Authors:** Yuki Togawa, Kyohei Kamihata, Yoshio Nagahisa, Kazuyuki Kawamoto

**Affiliations:** a Department of Surgery, Kurashiki Central Hospital, Kurashiki City, Okayama, Japan; b Department of Surgery, Osaka Red Cross Hospital, Osaka City, Osaka, Japan.

**Keywords:** FROGS, intestinal obstruction, obturator hernia, reduction

## Abstract

To evaluate the effectiveness of 4-hand reduction for obturator hernia with the guidance of sonography (FROGS) as a new treatment strategy for obturator hernia. Since November 2019, FROGS was performed for all patients with obturator hernia at our emergency department. We retrospectively compared the clinical data of 20 patients who underwent FROGS (after FROGS group) to those of 23 patients who did not (before FROGS group). All patients except one were female. The male-to-female ratio, age, duration of symptoms, lesion site, and predisposing factors did not significantly differ between groups. However, the diameter of the prolapsed bowel and the body mass index of the after FROGS group were significantly larger and lower, respectively. Manual reduction was successfully performed for all 20 patients in the after FROGS group, and bowel resection was avoided for all 20 cases. However, 14 patients in the before FROGS group underwent manual reduction; of these, only one was successfully treated using a method other than FROGS, and 8 patients underwent bowel resection. There were no significant differences between groups in terms of postprocedural complications or mortality within 30 days of hernia presentation. The FROGS technique was safe and reproducible and could be used as the first choice of treatment for patients with obturator hernia.

## 1. Introduction

Obturator hernias are rare forms of abdominal hernias that account for < 1% of all abdominal hernias.^[1]^ Previously, determining a definite preoperative diagnosis has been difficult; however, it has become much easier with the use of advanced imaging techniques.^[2–5]^ Obturator hernia usually develops in elderly, thin, and multiparous women who often have comorbidities and other debilitating conditions that can preclude them from undergoing emergency surgery. Therefore, the postoperative mortality rate after emergency surgery is high.^[6,7]^ However, after the diagnosis is determined, emergency surgery is usually performed because delaying treatment can lead to intestinal ischemia and subsequent perforation.^[1]^ Previous reports have described successful manual reductions of obturator hernias.^[8–11]^ However, these were case reports with no statistical confirmation of the efficacy of manual reduction. Therefore, until recently, there has been no controversy regarding whether manual reduction (with or without elective surgery) or emergency surgery was more effective. Kamihata et al^[12]^ described a new repositioning maneuver for incarcerated obturator hernia called 4-hand reduction for obturator hernia with the guidance of sonography (FROGS) in Japan. This simple technique was created based on previous reports to treat obturator hernias in the emergency department. To the best of our knowledge, this is the first report to statistically demonstrate the efficacy of a noninvasive manual reduction technique, FROGS, as a new treatment strategy for obturator hernias and includes 6 more cases than the previous study.^[12]^

## 2. Methods

### 2.1. Study design

The clinical data of all patients with an obturator hernia who were admitted to the Department of Surgery at the Kurashiki Central Hospital between April 2016 and December 2021 were retrospectively reviewed. We have implemented FROGS in the emergency department as the first choice of treatment since November 2019. We compared the clinical data (sex, age, body mass index, duration of symptoms, diameter of the prolapsed bowel, hernia site, predisposing factors, manual reduction success rate, surgery type, necessity of bowel resection, complications, hospital stay, outcome, and follow-up) of patients who underwent FROGS (after FROGS group) with those of patients who did not (before FROGS group). All procedures performed during this study were in accordance with the ethical standards of the institutional research committee (3658) and with the 1964 Helsinki Declaration and its later amendments or comparable ethical standards. Written informed consent was obtained for the academic use of photographs of the patients.

### 2.2. Steps used for FROGS

**Figure 1.**
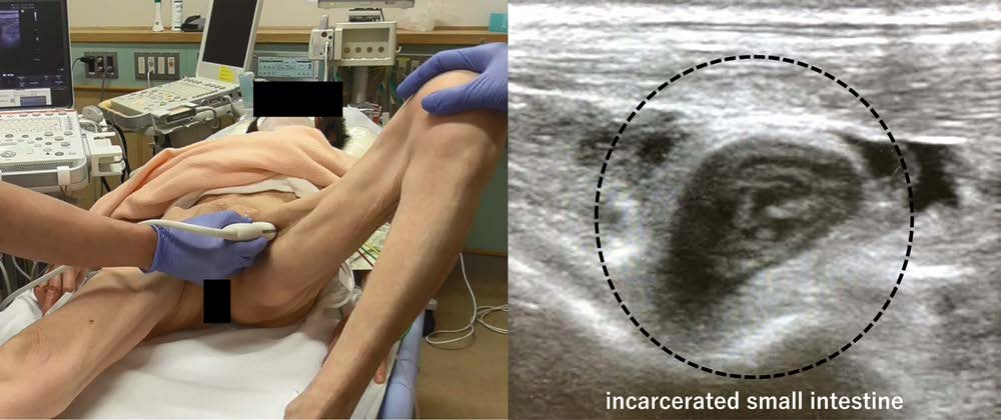
The surgeon locates and confirms the hernial sac between the adductor longus and the pubis. The assistant supports the patient to keep the hip joint flexed and rotated externally to ensure the hernial sac is well-visualized. The ultrasound image shows a dilated small intestine incarcerated in the hernial sac.

We created FROGS as a bedside reduction method for obturator hernias. It is performed using real-time sonographic guidance in the emergency department and is based on previous reports.^[8–11]^ This technique requires 2 surgeons, which is why it is referred to as the 4-hand technique. Before initiating this procedure, the presence of an obturator hernia (a hernial sac between the adductor longus and the pubis) must be confirmed. As the surgeon confirms the hernial sac, the assistant supports the patient to keep the hip joint flexed and rotated externally so that the hernial sac can be visualized (Fig. 1). Specifically, during FROGS, the following steps are performed:

*Step 1*: The surgeon places the transducer in the left hand on the groin area of the patient (cranial to the inguinal ligament) while the right hand pushes the hernial sac. The assistant places the hip and knee joints in the flexed position.

**Figure 2.**
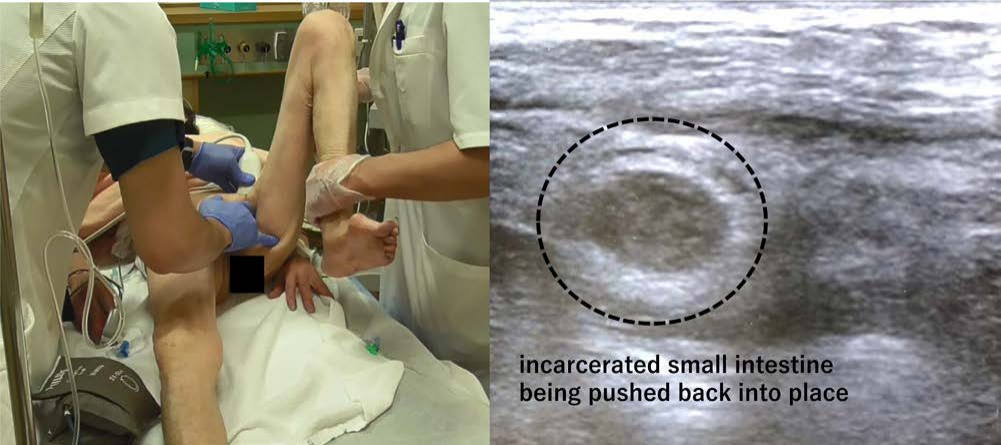
Step 1 and step 2. The surgeon places the transducer in the left hand on the groin area of the patient (just cranial to the inguinal ligament) while the right hand pushes the hernial sac. The assistant places the hip joint and the knee joint in a flexed position. Subsequently, the assistant slightly rotates the hip joint laterally, thus widely opening the hernial orifice. The ultrasound image shows the incarcerated small intestine being pushed back by the surgeon’s right hand.

*Step 2:* The assistant slightly rotates the hip joint laterally, thus widely opening the hernia orifice (Fig. 2).

**Figure 3.**
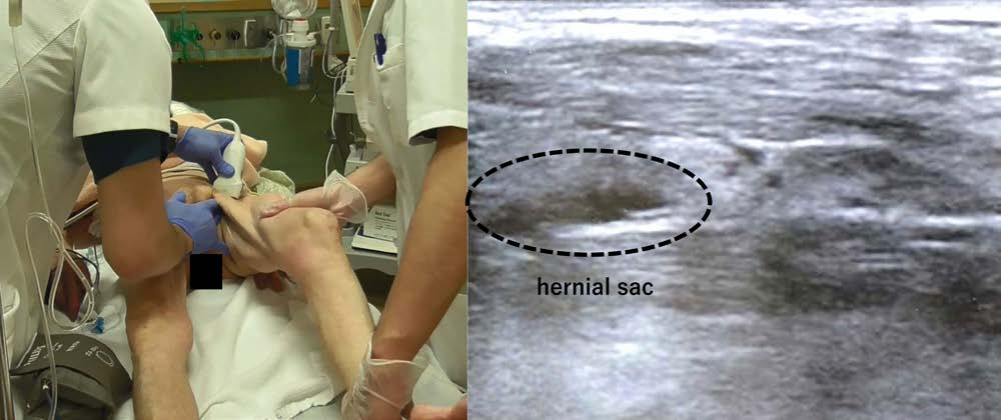
Step 3. The assistant places the hip and knee joints in an extended position. During this maneuver, the surgeon pushes the hernial sac more forcefully. The ultrasound image shows an empty hernial sac after this maneuver.

*Step 3:* The assistant extends the hip and knee joints (Fig. 3).

To facilitate these movements, the assistant places one hand on the upper thigh of the patient and the other hand on the lower thigh of the patient. While the assistant performs the 3-step maneuver, the surgeon continues to push the hernial sac. During step 3, the surgeon pushes the hernial sac more forcefully compared to that during the previous steps. The muscles along the obturator canal relax, allowing the hernial sac to shrink. The 2 physicians continue this maneuver repeatedly until the hernial sac is completely pushed back. This movement is reminiscent of a frog moving its legs while swimming, which is another reason why the method is called FROGS.

### 2.3. Statistical analyses

All statistical analyses were performed using R software version 4.0.3 (The R Project for Statistical Computing, Vienna, Austria). Continuous variables are expressed as the mean ± standard deviation, and categorical variables are expressed as absolute numbers and percentages. All variables were analyzed using Student’s *t* test and the χ^2^ test or Fisher’s exact test for continuous and categorical variables, respectively. *P* value < .05 was considered statistically significant.

## 3. Results

A total of 43 patients with obturator hernias were admitted to the Department of General Surgery at Kurashiki Central Hospital between April 2016 and December 2021. Before the FROGS technique was implemented, 23 patients had been admitted (before FROGS group). After November 2019, 20 patients were admitted; all of them underwent FROGS as the first treatment choice (after FROGS group). The demographic characteristics of all the patients are summarized in Table 1.

**Table 1:** Comparison of the 2 groups at the first visit.

	Before FROGS	After FROGS	*P* value
Patients (male)	23 (0)	20 (1)	0.278
Age	88.3 ± 5.53	87.1 ± 6.67	0.506
Body mass index	17.5 ± 2.73	16.1 ± 2.73	0.033
Duration of symptoms (d)	1.52 ± 1.59	0.95 ± 1.23	0.137
Prolapsed bowel (cm)	2.35 ± 0.51	2.58 ± 0.52	0.049
Lesion site (right/left)	13/10	8/12	0.279
Predisposing factors			0.429
Cardiopathy	8 (34.8%)	7 (35%)	
Vascular disease	5 (21.7%)	5 (25%)	
Hypertension	9 (39.1%)	5 (25%)	
Lung disease	3 (13.0%)	2 (10%)	
Diabetes mellitus	1 (4.3%)	2 (10%)	
Chronic kidney disease	4 (17.4%)	0 (0%)	
Dementia	1 (4.3%)	3(15%)	
Manual reduction (successful/attempted)	1/14 (7.14%)	20/20 (100%)	<0.001
Patients who underwent emergency surgery	21/23 (91.3%)	0/20 (0%)	<0.001

FROGS = 4-hand reduction for obturator hernia with the guidance of sonography.

All patients but one were female. All 43 patients were diagnosed with obturator hernia preoperatively by computed tomography. There were no significant differences in the male-to-female ratio, age, duration of symptoms, lesion site, and predisposing factors between groups. However, the body mass index was significantly lower (*P* = .033) (Table 1) and the diameter of the prolapsed bowel was significantly larger in the after FROGS group (*P* = .049) (Table 1).

Manual reduction was successfully performed for all 20 patients in the after FROGS group (success rate of 100%). In the before FROGS group, 14 patients underwent manual reduction; of them, only one was successfully treated (success rate of 7.14%) using a method other than FROGS. The success rate of manual reduction was significantly higher in the after FROGS group than in the before FROGS group (*P* < .001) (Table 1). The number of patients who underwent emergency surgery was significantly smaller in the after FROGS group than in the before FROGS group (*P* < .001) (Table 1).

Table 2 shows the number of chosen therapeutic methods for both groups. Of the 20 patients in the after FROGS group, 6 underwent elective surgery; all of these surgeries were performed using the transabdominal preperitoneal (TAPP) technique. Fourteen patients did not undergo elective surgery because of comorbidities or because their family did not consent to the procedure (nonoperative management). In the before FROGS group, successful manual reduction was performed for only one patient who underwent elective surgery performed using TAPP repair. Emergency surgery could not be performed for one patient because of severe heart failure; this patient died 6 days after admission. The other 21 patients underwent emergency surgery (17 underwent laparotomy and 4 underwent TAPP repair).

**Table 2:** Chosen therapeutic methods.

	Before FROGS	After FROGS
All surgery (TAPP/inguinal approach or laparotomy)	22 (5/17)	6 (6/0)
Emergency surgery (TAPP/inguinal approach or laparotomy)	21 (4/17)	0 (0/0)
Elective surgery (TAPP/inguinal approach or laparotomy)	1 (1/0)	6 (6/0)
Nonoperative management	1	14

FROGS = 4-hand reduction for obturator hernia with the guidance of sonography, TAPP = transabdominal preperitoneal.

All 7 elective surgeries performed in both groups involved laparoscopic repair (TAPP repair). However, of the 21 patients who underwent emergency surgeries, only 4 (19.0%) underwent laparoscopic TAPP repair. The rate of TAPP repair was significantly higher for patients who underwent elective surgery (*P* < .001) (Table 3).

**Table 3:** Comparison of elective surgery and emergency surgery as operative methods.

	Elective surgery	Emergency surgery	*P* value
TAPP/inguinal approach or laparotomy	7/0	4/17	<0.001

FROGS = 4-hand reduction for obturator hernia with the guidance of sonography, TAPP = transabdominal preperitoneal.

Bowel resection was avoided for all 20 patients in the after FROGS group; however, 8 patients in the before FROGS group underwent bowel resection. The bowel resection rate was significantly lower in the after FROGS group than in the before FROGS group (*P* = .003) (Table 4). The length of hospitalization was significantly shorter in the after FROGS group (*P* = .028) (Table 4) There were no significant differences between groups in terms of postprocedural complications (*P* = .645) (Table 4) or mortality rates within 30 days of hernia presentation (*P* = .177) (Table 4).

**Table 4:** Between-group comparisons of operative and postprocedural data.

	Before FROGS	After FROGS	*P* value
Bowel resection	8 (34.8%)	0 (0%)	0.003
Postprocedural complication			0.645
Aspiration pneumoniae	3 (13.0%)	2 (10%)	
Urinary tract infection	2 (8.6%)	0 (0%)	
Intra-abdominal abscess	2 (8.6%)	0 (0%)	
Heart failure	1 (4.3%)	0 (0%)	
Ileus	1 (4.3%)	0 (0%)	
False reduction	1 (4.3%)	0 (0%)	
Death within 30 d after admission	2 (9.5%)	0 (0%)	0.177
Hospital stay (d)	13 ± 11.8	8.3 ± 9.75	0.028

FROGS = 4-hand reduction for obturator hernia with the guidance of sonography.

## 4. Discussion

We statistically evaluated the effectiveness of FROGS as a new treatment strategy for obturator hernia. Currently, manual reduction with FROGS has a success rate of 100%. The results showed that patients can avoid emergency surgery and bowel reduction without increasing the risk of postprocedural complications or mortality within 30 days of hernia presentation because of FROGS.

Obturator hernia is a rare type of abdominal hernia caused by the loss of preperitoneal fat and lymphatic tissues that normally overlie the obturator canal and results in a space around the obturator vessels and nerves.^[13]^ Concomitant illnesses, such as chronic obstructive pulmonary disease, constipation, and kyphoscoliosis, could result in increased intraperitoneal pressure and facilitate the growth of the hernial sac.^[14]^ Lean and elderly women are usually affected because they have less preperitoneal fat and several comorbidities that favor the pathogenesis of this condition.

Laparotomy was once the standard approach for the treatment of obturator hernia because the preoperative diagnosis is difficult because of its rarity and the signs and symptoms are nonspecific. Ziegler et al^[15]^ mentioned that “an obturator hernia needs a laparotomy, not a diagnosis.” Many surgeons believe that emergency laparotomy is the optimal treatment for possible incarcerated obturator hernias. However, recent advances in imaging techniques, such as computed tomography and ultrasonography, have improved the correct preoperative diagnosis rate, thus making less invasive approaches feasible.^[2–4]^ Some studies have described less invasive therapeutic strategies, such as elective surgery after successful manual reduction.^[5–7]^ In these studies, elective surgeries were demonstrated to be associated with a higher rate of TAPP repair implementation. The decision to undertake laparoscopic approaches less frequently during emergency surgeries could be attributed to the smaller peritoneal cavity resulting from a dilated intestine.

This study aimed to evaluate the efficacy of FROGS as the first choice of treatment for incarcerated obturator hernias. Patients with an obturator hernia often have comorbidities, thus making them unsuitable for emergency surgeries. They should be treated with less invasive therapy in an elective setting, if possible. Additionally, some patients and their families do not consent to surgery. Ceresoli et al^[16]^ showed that emergency surgery for complicated inguinal hernias is burdened by high morbidity and mortality rates for elderly patients. For patients with asymptomatic or minimally symptomatic inguinal hernias, watchful waiting is recognized as an acceptable option.^[17,18]^ Although these studies have mainly focused on inguinal hernias, the results could be applied to obturator hernias as well.

During our study, emergency surgeries for asymptomatic or minimally symptomatic patients were avoided because of the implementation of FROGS. Some of these patients underwent safe elective TAPP repair; however, others did not because of comorbidities or family wishes. Irrespective of whether elective surgery was performed, bowel resection was avoided for all patients. Additionally, the short-term prognoses for the after FROGS and before FROGS groups were comparable, and FROGS facilitated a shorter hospital stay. Although the balance between the risks of elective surgery and the risks of the watchful approach is still a topic of debate in the absence of specific recommendations for elderly patients,^[16]^ a watchful approach could be a choice based on these results and the literature.

Although patients who do not undergo elective surgery are still at risk for recurrence, FROGS can be reproducible in cases of recurrence because of its high success rate. Therefore, we believe that FROGS could be the first choice of treatment for any case of obturator hernia incarceration. The mechanism of FROGS is unclear and may be complex. We believe that the complex coordinated movements of the muscles around the obturator canal may have an important role. The driving pressure generated by these movements helps surgeons to reduce the hernial sac. Furthermore, at some point during this maneuver, the obturator canal may be maximally relaxed (usually during step 3, based on our experience). By repeating FROGS, we can eventually find this particular point.

It is difficult to decide whether bowel resection is necessary, especially if FROGS is implemented. No patient in the after FROGS group required a bowel resection. The longest duration from symptom onset to hernia presentation was 72 hours. Based on these results, we can argue that if there is no obvious evidence of strangulation or ischemia, manual reduction using FROGS is acceptable within 72 hours.

The limitation of this study is its relatively small sample size. Further observations and analyses are necessary to confirm the effectiveness of FROGS. Nevertheless, manual reduction with FROGS was found to be safe and reproducible and can be the first choice of treatment for patients with obturator hernias. FROGS can be used instead of emergency surgery as a less invasive method to treat patients with obturator hernias while preserving the bowel.

## 5. Conclusion

Based on the retrospective data of 43 patients, which included those of 20 patients who underwent FROGS (after FROGS group) and 23 patients who did not (before FROGS group), FROGS was found to be safe and reproducible and can be the first treatment choice for obturator hernias instead of emergency surgery. More observations and analyses are necessary to further discuss the scope of the indications for FROGS because the sample size was relatively small. However, we believe our results are quite promising.

## Acknowledgments

We would like to thank Editage for English proofreading.

## Author contributions

All authors contributed to the study conception and design. YT and KK performed the material preparation, data collection, and data analysis. YT wrote the first draft of the manuscript. All authors commented on previous versions of the manuscript. All authors read and approved the final manuscript.

**Conceptualization:** Yoshio Nagahisa, Kazuyuki Kawamoto.

**Writing – original draft:** Yuki Togawa.

**Writing – review and editing:** Kyohei Kamihata.

**Writing – review and editing:** Yoshio Nagahisa

**Writing – review and editing:** Yuki Togawa.
